# Control of Grain Weight and Size in Rice (*Oryza sativa* L.) by *OsPUB3* Encoding a U-Box E3 Ubiquitin Ligase

**DOI:** 10.1186/s12284-022-00604-1

**Published:** 2022-11-23

**Authors:** Shi-Lin Wang, Zhen-Hua Zhang, Ye-Yang Fan, De-Run Huang, Yao-Long Yang, Jie-Yun Zhuang, Yu-Jun Zhu

**Affiliations:** 1 State Key Laboratory of Rice Biology, China National Rice Research Institute, Hangzhou, 310006 China; 2 National Nanfan Research Institute (Sanya), Chinese Academy of Agricultural Sciences, Sanya, 572024 China

**Keywords:** Rice, Grain weight, Grain size, Milling quality, Ubiquitin-ligase enzyme, U-box domain

## Abstract

Grain weight and size, mostly determined by grain length, width and thickness, are crucial traits affecting grain quality and yield in rice. A quantitative trait locus controlling grain length and width in rice, *qGS1-35.2*, was previously fine-mapped in a 57.7-kb region on the long arm of chromosome 1. In this study, *OsPUB3*, a gene encoding a U-box E3 ubiquitin ligase, was validated as the causal gene for *qGS1-35.2*. The effects were confirmed firstly by using CRISPR/Cas9-based mutagenesis and then through transgenic complementation of a Cas9-free knock-out (KO) mutant. Two homozygous KO lines were produced, each having a 1-bp insertion in *OsPUB3* which caused frameshift mutation and premature termination. Compared with the recipient and a transgenic-negative control, both mutants showed significant decreases in grain weight and size. In transgenic complementation populations derived from four independent T_0_ plants, grain weight of transgenic-positive plants was significantly higher than transgenic-negative plants, coming with increased grain length and a less significant decrease in grain width. Based on data documented in RiceVarMap V2.0, eight haplotypes were classified according to six single-nucleotide polymorphisms (SNPs) found in the *OsPUB3* coding region of 4695 rice accessions. Significant differences on grain size traits were detected between the three major haplotypes, Hap1, Hap2 and Hap3 that jointly occupy 98.6% of the accessions. Hap3 having the largest grain weight and grain length but intermediate grain width exhibits a potential for simultaneously improving grain yield and quality. In another set of 257 *indica* rice cultivars tested in our study, Hap1 and Hap2 remained to be the two largest groups. Their differences on grain weight and size were significant in the background of non-functional *gse5*, but non-significant in the background of functional *GSE5*, indicating a genetic interaction between *OsPUB3* and *GSE5*. Cloning of *OsPUB3* provides a new gene resource for investigating the regulation of grain weight and size.

**Supplementary Information:**

The online version contains supplementary material available at 10.1186/s12284-022-00604-1.

## Background

Rice (*Oryza sativa* L.) is one of the most important food crops in the world. Simultaneous improvement of rice grain yield and quality remains a difficult task for rice breeders as grain yield and quality are often negatively correlated with each other (Sakamoto et al. 2008). Grain weight and size, mostly determined by grain length, width and thickness, are crucial traits affecting grain quality and yield in rice (Zuo et al. 2014). Understanding the genetic and molecular basis for natural variation of grain weight and size is important for developing rice varieties with high yield and superior quality.

To date, causal genes for 25 quantitative trait loci (QTLs) regulating grain weight and size in rice have been cloned. They are located on all rice chromosomes except chromosomes 4, 11 and 12. The number of genes located on chromosome 3 is the largest, with a total of eight genes. They are *OsLG3* (Yu et al. 2017), *OsLG3b*/*qLGY3* (Liu et al. 2018; Yu et al. 2018), *GS3.1* (Zhang et al. 2021), *GS3* (Fan et al. 2006), *SG3* (Li et al. 2020), *GL3.1* (Qi et al. 2012), *GSA1* (Dong et al. 2020) and *qTGW3* (Hu et al. 2018). Among them, *OsLG3*, *OsLG3b*/*qLGY3* and *GS3.1* are linked in the 4.3-Mb to 6.9-Mb region on the short arm, *GS3* and *SG3* are tightly linked in the 16.7-Mb to 16.9-Mb pericentromeric region, and the other three genes are located on the long arm. Four genes, *GW6* (Shi et al. 2020), *TGW6* (Ishimaru et al. 2013), *GW6a* (Song et al. 2015) and *GL6* (Wang et al. 2019a), are located on chromosome 6, of which *GW6* is located on the short arm and the other three are closely linked in the 26.0-Mb to 27.6-Mb region on the long arm. Followed are three genes on two chromosomes, including *GW2* (Song et al. 2007), *GS2*/*GL2*/*GLW2* (Hu et al. 2015; Che et al. 2016; Li et al. 2016) and *TGW2* (Ruan et al. 2020) on chromosome 2, and *GS5* (Li et al. 2011), *GW5*/*GSE5* (Duan et al. 2017; Liu et al. 2017) and *qGL5* (Qiao et al. 2021) on chromosome 5. Then are two genes on two chromosomes, including *GLW7* (Si et al. 2016) and *GL7*/*GW7* (Wang et al. 2015a, 2015b) on chromosome 7, and *GW10* (Zhan et al. 2021) and *GL10*/*OsMADS56* (Zhan et al. 2022; Zuo et al. 2022) on chromosome 10. The remaining three are *qTGW1.2b* (Chan et al. 2021), *GW8* (Wang et al. 2012) and *GS9* (Zhao et al. 2018) on chromosomes 1, 8 and 9, respectively. Of these, *GS3* and *GW5* were earlier isolated as major QTLs controlling grain length and grain width, respectively (Fan et al. 2006; Weng et al. 2008). More recently, *GSE5* was confirmed as the causal gene for *GW5* (Duan et al. 2017; Liu et al. 2017).

Rice grain quality parameters are usually classified into four categories, *i.e.* appearance quality, milling quality, cooking and eating quality, and nutritional quality. The appearance and milling quality are closely related to the grain size traits (Xie et al. 2013; Bao et al. 2019). Among the 25 cloned QTLs for grain size, pleiotropic effects with unfavorable association between grain yield and appearance and/or milling quality have been reported. The *gw2*^WY3^ allele showed positive effects on grain yield by increasing grain width (GW) and thousand grain weight (TGW), but had negative effects on grain quality by increasing chalky rice percentage (CRP) and decreasing brown rice percentage and milled rice percentage (Song et al. 2007). The *GS2*^BDL^, *tgw2*^93−11^ and *GLW7*^LGH^ alleles are favorable for increasing grain length (GL), GW and TGW, but unfavorable for enlarging CRP (Hu et al. 2015; Si et al. 2016; Ruan et al. 2020). The *GW8*^HJX74^ allele is favorable for increasing GW and TGW but unfavorable for decreasing endosperm transparency and increasing CRP (Wang et al. 2015a).

Ubiquitination is a type of post-translational modification of proteins, widely occurring in eukaryotic cells. It plays critical roles in controlling the synthesis and degradation of proteins in plant growth, development and responses to biotic and abiotic stresses. The ubiquitination system includes a cascade of enzymatic reactions determining by three main enzymes: ubiquitin-activating enzyme (E1), ubiquitin-conjugating enzyme (E2), and ubiquitin-ligase enzyme (E3). The E3 ligase can be divided into two classes: the Homologous to E6-associated protein Carboxyl Terminus type and the Really Interesting New Gene (RING)/U-box type (Mao et al. 2022). *GW2* encoding a RING-type protein with E3 ubiquitin ligase activity showed a major effect on grain size (Song et al. 2007). In addition, 77 annotated genes for U-box type E3 ligases of eight groups were found in rice genome (Zeng et al. 2008).

In our previous study, one QTL, *qGS1-35.2* controlling GL and GW difference between *indica* rice varieties Zhenshan 97 (ZS97) and Milyang 46 (MY46), was fine-mapped in a 57.7-kb region one the long arm of chromosome 1 (Dong et al. 2018). In this study, the most likely gene for *qGS1-35.2*, *Os01g0823900* encoding the U-box E3 ubiquitin ligase OsPUB3, was validated using CRISPR/Cas9-based gene knock-out (KO) and transgenic complementation of the KO mutant. Knock-out of *OsPUB3* caused a decrease in grain weight and size but an increase in head rice. Correspondingly, transformation of a functional *OsPUB3* allele into the KO mutant resulted in increased grain weight and size. Our finding provides a new gene resource to improve grain size and quality.

## Results

### Genetic Effects of *OsPUB3* Identified Using CRISPR/Cas9-Based Mutagenesis

**Fig. 1:**
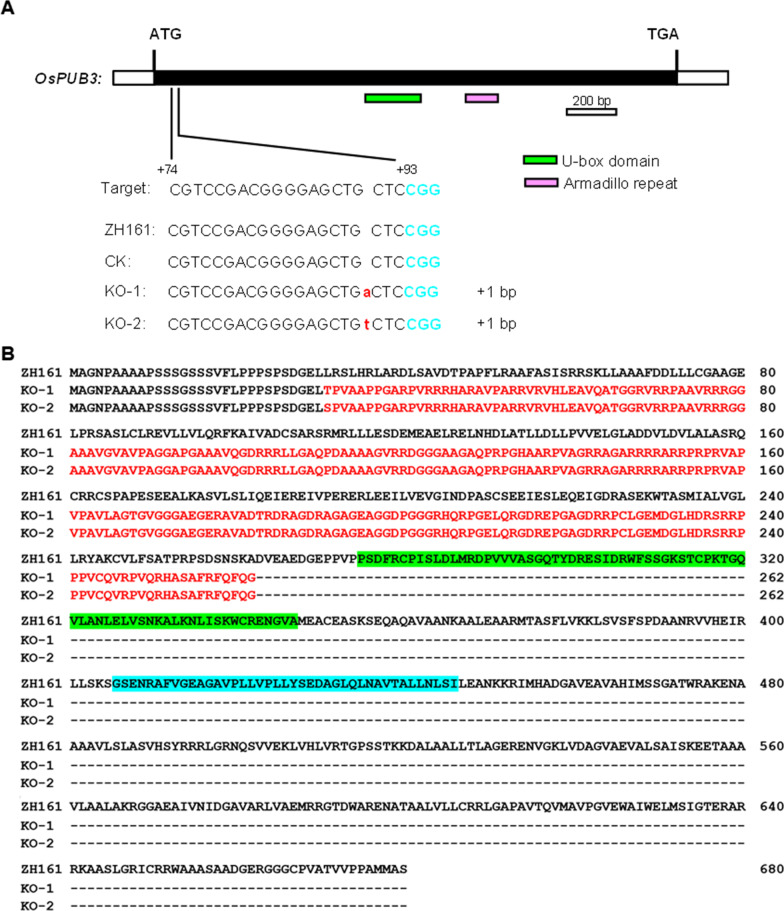
Variations of the *OsPUB3* knock-out mutants. **A** Variations of the DNA sequences in the target region. The protospacer adjacent motif site is shown in blue. Insertion is indicated by lowercase letter in red. **B** Variations of the predicted amino acid sequences. Letters in red indicate amino acid sequences of frameshift mutation. U-box domain is in green. Armadillo repeat domain is in blue. ZH161 is the recipient. CK is the transgenic-negative control. KO-1 and KO-2 are two homozygous knock-out mutants

*OsPUB3* KO mutants were produced for validating the effects of *OsPUB3*. Using the CRISPR/Cas9 system, two independent T_0_ homozygous mutants (KO-1 and KO-2) were produced from the recipient Zhonghui 161 (ZH161), an *indica* rice cultivar. Both the mutants had a 1-bp insertion at + 91 in the coding region (Fig. 1A). The sequence variation introduced a frameshift mutation at the 31st amino acid (AA) and led to a premature translation termination at the 263rd AA. Finally, the number of amino acid residues changed from 680 to 262 for the two mutants (Fig. 1B).

**Fig. 2:**
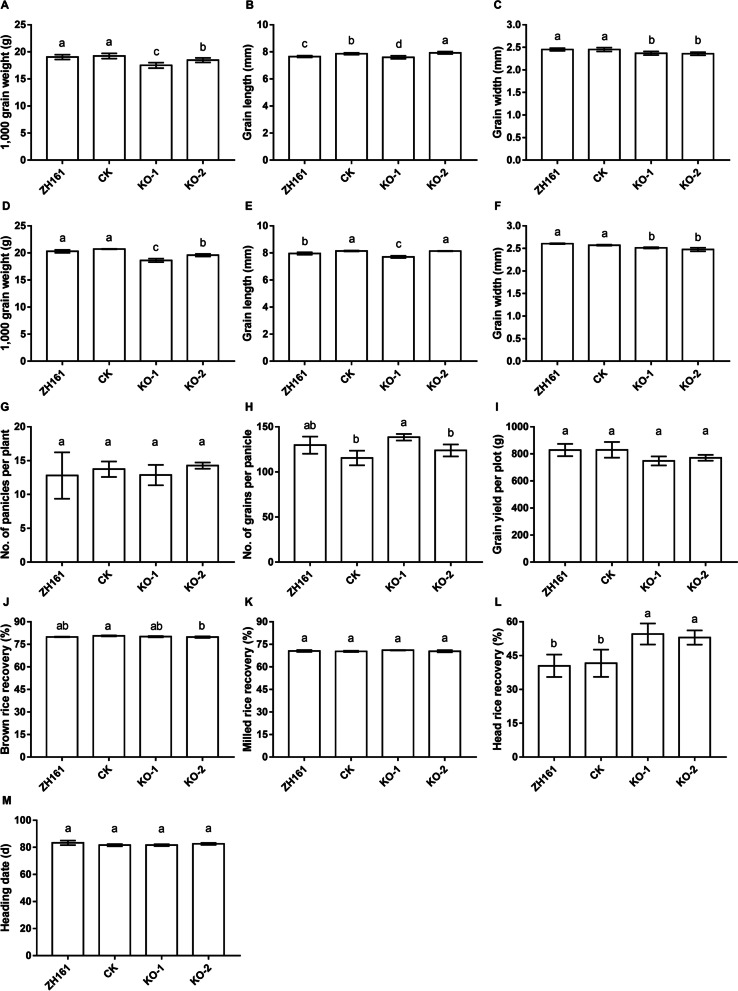
Phenotypic change due to *OsPUB3* knock-out. **A**–**C** Three traits measured in the T_1_ experiment using 20 plants. **D**–**M** Ten traits measured in the T_2_ experiment using a randomized complete block design with three replications. The Duncan’s multiple range test was employed to test the phenotypic differences among the knock-out mutants, transgenic-negative control and recipient. Any two groups having a common letter, are not significantly different at a 0.05 level of significance. Instead, any two groups with different letters are significantly different at a 0.05 level of significance

T_1_ and T_2_ populations of the two mutants were tested against the recipient ZH161 and a transgenic negative control (CK). The T_1_ trial was conducted in 2019, in which 20 plants of each population were measured for TGW, GL and GW. The T_2_ trial was conducted in 2021, using a randomized complete block design with three replications. A total of ten traits were measured, including TGW, GL, GW, number of panicles per plant (NP), number of grains per panicle (NGP), grain yield per plot (GY), brown rice recovery (BRR), milled rice recovery (MRR), head rice recovery (HRR), and heading date (HD). Phenotypic differences among the four genotypes were analyzed using the Duncan’s multiple range test. The results are presented in Fig. 2 and Additional file 2: Table S1.

Compared with ZH161 and CK, the two mutants showed significant decreases in TGW and GW in both the T_1_ (Fig. 2A, C) and T_2_ (Fig. 2D, F) trials. Meanwhile, non-significant differences were observed between ZH161 and CK. For TGW, decreases over CK in T_1_ and T_2_ were 1.73 g (9.0%) and 2.09 g (10.1%) in KO-1, and 0.76 g (3.9%) and 1.13 g (5.4%) in KO-2, respectively (Additional file 2: Table S1). For GW, decreases over CK in T_1_ and T_2_ were 0.083 mm (3.4%) and 0.059 mm (2.3%) in KO-1, and 0.093 mm (3.8%) and 0.095 mm (3.7%) in KO-2, respectively. Decreases of TGW and GW due to *OsPUB3* knock-out were evident, although the effect found in KO-1 was much stronger on TGW and slightly weaker on GW than that detected in KO-2.

Results on GL were much less consistent. Significant differences were observed between the two controls themselves, and changes over the controls varied greatly between KO-1 and KO-2. KO-1 remained to significantly decrease over ZH161 and CK in both the T_1_ (Fig. 2B) and T_2_ (Fig. 2E) trials. The decreases over CK were 0.265 mm (3.4%) in T_1_ and 0.433 mm (5.3%) in T_2_ (Additional file 2: Table S1). In terms of the percentage change, KO-1 had a greater decrease in GL than in GW. On the other hand, KO-2 had a significant increase over CK in T_1_ (Fig. 2B) with a small value of 0.070 mm (0.9%), and a non-significant change in T_2_ (Fig. 2E). It could be concluded that knock-out of *OsPUB3* caused a clear effect for decreasing grain weight, but the effect on the two component traits, grain length and width, was more variable.

To investigate whether cell proliferation or expansion is the source for the changes in grain weight and size, T_2_ samples of CK and KO-1 was measured and compared for the length, width and number of the outer glume epidermal cells. Non-significant difference was detected on the cell length and width, but the cell numbers in both the longitudinal and transverse directions were higher in CK than in KO-1 (Additional file 1: Figure S1). These results indicate that *OsPUB3* affects grain length and width by controlling cell division.

Among the seven traits that were only tested in the T_2_ trial, significant differences among genotypes were detected on three traits, including NGP, BRR and HRR. For NGP, significant difference was only detected between KO-1 and CK (Fig. 2H), with grain number decreased by 22.9 in KO-1. For BRR, significant difference was only detected between KO-2 and CK (Fig. 2J), with the trait value decreased by 0.8% in KO-2. For HRR, the two mutants showed significant increases over ZH161 and CK. Compared with CK, the trait values in KO-1 and KO-2 increased by 13.0% and 11.4%, respectively. Non-significant difference was detected on the remaining four traits, including NP, GY, MRR and HD (Fig. 2G, I, K and M).

These results indicate that *OsPUB3* is the causal gene for *qGS1-35.2*. This gene had significant genetic effects on grain size, and simultaneously influenced the critical trait of rice milling quality, HRR. Changes in grain size traits and HRR due to *OsPUB3* knock-out are obvious (Additional file 1: Figure S2).

### Genetic Effects of *OsPUB3* Identified Using Transgenic Complementation

Genetic effects of *OsPUB3* on grain size were further tested using transgenic complementation of a knock-out mutant. As described in the knock-out trial, the effect on grain size due to *OsPUB3* knock-out was larger and more stable in KO-1 than in KO-2. Thus, KO-1 was targeted for transgenic complementation. A Cas9-free mutant, TC3-6, was selected from progenies of KO-1 and transformed with the *OsPUB3* gene isolated from MY46, the male parent of the original mapping population. Four independent T_0_ transformants were identified and the resulting T_1_ populations were tested in 2021. Results on the phenotypic differences between transgenic-negative and transgenic-positive plants, tested by using the Student’s *t*-test, are presented in Table 1.

**Table 1:** Genetic effects of *OsPUB3* in four transgenic complementation populations

Name	No. of plants		Trait^a^	Mean ± SD		± (P-N)^b^	± (P-N)%^c^
	Negative	Positive		Negative	Positive		
CP-1	19	89	TGW	18.16 ± 0.86	18.79 ± 0.99	0.63^**^	3.5
			GL	7.659 ± 0.150	7.747 ± 0.196	0.088^*^	1.2
			GW	2.494 ± 0.040	2.467 ± 0.035	− 0.027^**^	− 1.1
CP-2	28	69	TGW	17.47 ± 0.68	18.41 ± 0.72	0.95^****^	5.4
			GL	7.461 ± 0.159	7.611 ± 0.151	0.150^****^	2.0
			GW	2.487 ± 0.035	2.472 ± 0.052	− 0.014	− 0.6
CP-3	21	69	TGW	18.62 ± 0.54	19.53 ± 0.83	0.91^****^	4.9
			GL	7.806 ± 0.144	7.912 ± 0.171	0.107^**^	1.4
			GW	2.484 ± 0.029	2.444 ± 0.047	− 0.040^***^	− 1.6
CP-4	25	70	TGW	19.27 ± 0.85	19.97 ± 0.78	0.70^***^	3.6
			GL	7.911 ± 0.194	8.014 ± 0.183	0.103^**^	1.3
			GW	2.510 ± 0.039	2.471 ± 0.053	− 0.039^***^	− 1.6

^a^TGW, 1000-grain weight (g); GL, grain length (mm); GW, grain width (mm)

^b^Increase or decrease of transgenic-positive plants over transgenic-negative plants

^**c**^Percentage increase or decrease of transgenic-positive plants over transgenic-negative plants

*p < 0.05; **p < 0.01; ***p < 0.001; ****p < 0.0001

In each population, transgenic-positive plants were higher in TGW and GL compared with transgenic-negative plants. Changes in the trait values were also similar among the four population, ranging as 0.63–0.95 g (3.5–5.4%) for TGW, and 0.088–0.150 mm (1.2–2.0%) for GL. For GW, changes in populations CP-1, CP-3 and CP-4 were significant, with the positive plants decreased by 0.027–0.040 mm (1.1–1.6%) compared with the negative plants. In CP-2, the positive group also showed decreased GW compared with the negative group, but the difference was statistically non-significant.

### Allelic Variation of *OsPUB3* and Its Association with Grain Size

To analyze the allelic variation of *OsPUB3* in rice germplasm accessions, we search the sequence variations in the coding region of *OsPUB3* using the RiceVarMap V2.0 system (Zhao et al. 2015, 2021). Six single-nucleotide polymorphisms (SNPs) were found in the 4695 rice accessions including 2751 *Indica*, 1498 *Japonica*, 177 intermediate and 269 *Aus*. Eight haplotypes were classified and ordered following the number of accessions contained (Additional file 2: Table S2). The number of accessions included in Hap1, Hap2 and Hap3 accounted for 64.5%, 21.7% and 12.4% of the total, respectively, summing up to 98.6%. Based on our sequencing data of the three rice cultivars used in the map-based cloning of *OsPUB3*, ZS97 has Hap2, and MY46 and ZH161 have Hap1.

Then, we downloaded phenotypic data of TGW, GL, GW and ratio of GL to GW (RLW) documented in RiceVarMap V2.0. For haplotypes 4 to 8, the number of accessions having phenotypic data in each haplotype was less than 5, so we only compared phenotypic differences among Hap1, Hap2 and Hap3. As shown in Additional file 1: Figure S3, significant differences were detected on all the four traits. For TGW, the difference between Hap2 and Hap3 was non-significant, but they showed significant increase over Hap1. For GL, the three haplotypes were all significantly different, ranking from high to low as Hap3, Hap1 and Hap2. For GW, the three haplotypes were also all significantly different, ranking from high to low as Hap2, Hap3 and Hap1. For RLW, the difference between Hap1 and Hap3 was non-significant, but they showed significant increase over Hap2. Regarding MY46-type (Hap1) and ZS97-type (Hap 2) significant differences were observed on all the four traits. Compared with Hap2, Hap1 was lower in TGW and GW but higher in GL and RLW.

Since both ZS97 and MY46 are typical *indica* rice varieties, we further analyzed allelic variation of *OsPUB3* in 257 *indica* rice cultivars selected from the National Mid-term Genebank for Rice at the China National Rice Research Institute (Additional file 2: Table S3). They contained 119 improved varieties and 53 landraces in China and 85 cultivars introduced from other countries. Four of the haplotypes mentioned above, from Hap1 to Hap4, were identified in these varieties (Additional file 2: Table S2). The largest and second largest groups remained to be the MY46-type (Hap1) and ZS97-type (Hap 2), containing 230 (89.5%) and 19 varieties (7.4%), respectively. The remaining two haplotypes had a total of 8 varieties.

**Fig. 3:**
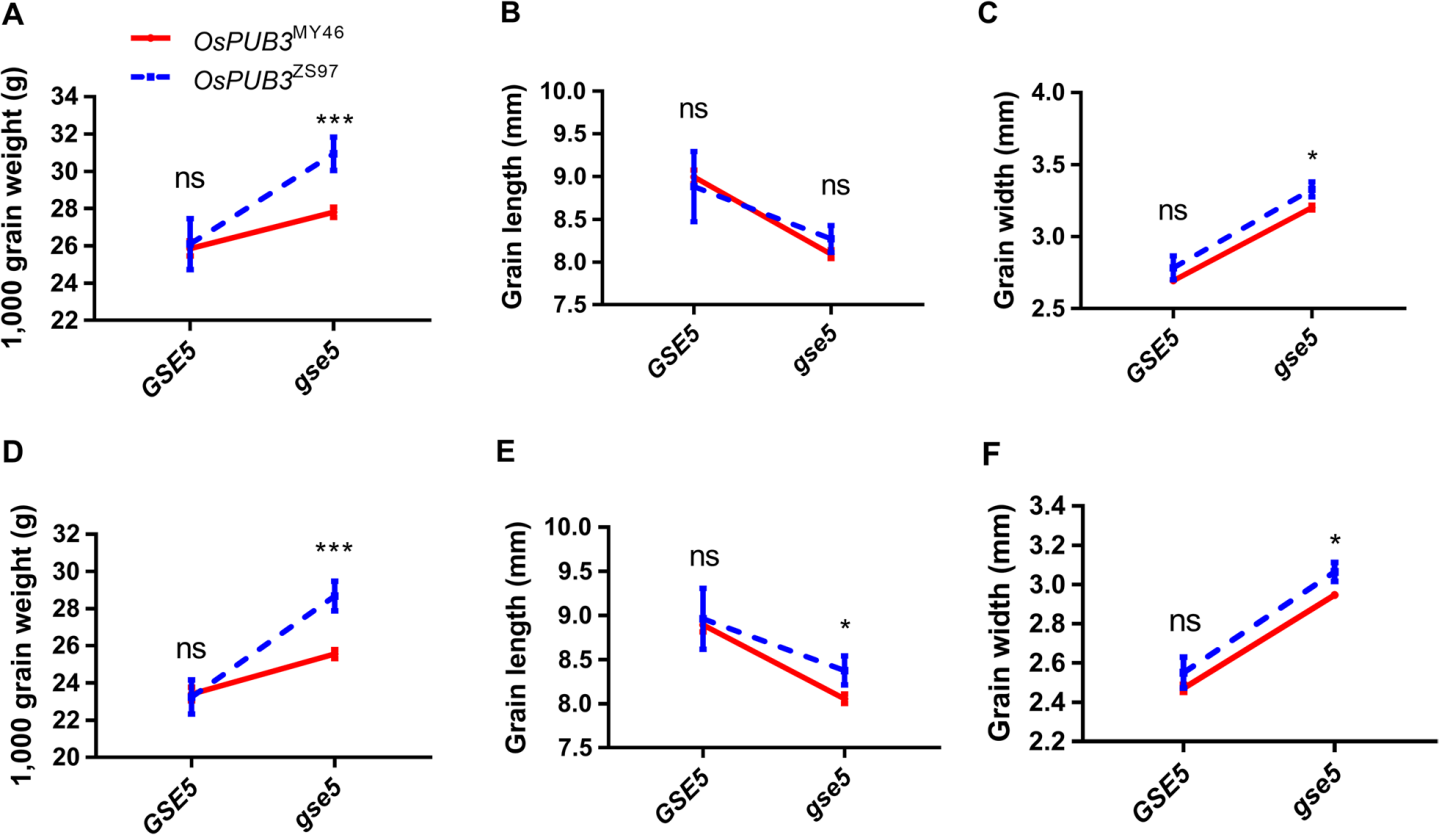
The effects of *OsPUB3* on three grain-size traits in the background of functional *GSE5* and non-functional *gse5*, respectively. **A**–**C** Three traits measured in Lingshui trial. **D**–**F** Three traits measured in Hangzhou trial. Values are given as the mean ± sem (*GSE5* background: *n* = 110 for *OsPUB3*^MY46^, *n* = 7 for *OsPUB3*^ZS97^; *ges5* background: *n* = 120 for *OsPUB3*^MY46^, *n* = 12 for *OsPUB3*^ZS97^). *p < 0.05; ***p < 0.001; *ns* not significant

The 257 varieties were grown in Lingshui (LS) and Hangzhou (HZ). Three traits, TGW, GL and GW, were measured. Phenotypic differences between the two haplotypes were tested using the Student’s *t*-test. In both the LS and HZ trails, TGW and GW were significantly higher in ZS97-type than in MY46-type, but the differences on GL were not significant (Additional file 1: Figure S4). This result indicates that the effect of *OsPUB3* on TGW is mainly through increasing GW. Among the cloned grain size genes, *GSE5* is a major gene controlling grain width, so we analyzed the effects of *OsPUB3* in the background of functional *GSE5* and non-functional *gse5*, respectively. As shown in Fig. 3, the effects of *OsPUB3* on TGW and GW were significant in the *gse5* background but non-significant in the *GSE5* background. This result indicates that there is a genetic interaction between *OsPUB3* and *GSE5*. The *OsPUB3* showed larger effects on grain size in the non-functional *gse5* background.

## Discussion

In this study, we confirmed that *OsPUB3* is the causal gene underlying a QTL for grain weight and size in rice. The effects were confirmed firstly by using CRISPR/Cas9-based mutagenesis and then through transgenic complementation of a Cas9-free KO mutant. One-bp insertion in *OsPUB3* caused frameshift mutation and premature termination, bringing about decreases in grain weight and size. Transgenic complementation of *OsPUB3* resulted in increased grain weight that came with increased grain length and a less significant decrease in grain width. Decreased grain weight and size owing to gene knock-out is accordance with increased grain weight and size due to genetic complementation, indicating that *OsPUB3* is a gene positively regulating grain weight and size in rice.

In this study, *OsPUB3* shows a clear influence on TGW in both the knock-out and complementation experiments. However, the control of *OsPUB3* on GL and GW is not clear. For GL, KO-1 significantly decreased, but KO-2 showed a significant increase or non-significant change, and a significant difference between ZH161 and CK was also found. For GW, both KO-1 and KO-2 showed significant decreases, but the transgenic complementation plants showed a less significant decrease rather than increase. To investigate possible influences of the genetic background, these four materials were examined by using whole-genome re-sequencing. Variations were found in some annotated genes, but none of them was found either in the cloned genes for grain weight and size or in the off-target loci predicted by the CRISPR-GE (http://skl.scau.edu.cn). More work is required to determine whether the variation observed on the effect of *OsPUB3* is related to interactions between *OsPUB3* and other background genes.

Genetic effects of minor genes traits are easily disturbed by major genes. For example, one minor gene, *SG3*, had a substantial effect on grain length and weight only when the major gene *GS3* is not functional (Li et al. 2020). A similar result was found in our study on *OsPUB3* and *GSE5*. The differences on grain weight and size between the two parental haplotypes of *OsPUB3* were significantly in the background of non-functional *gse5*, but non-significant in the background of functional *GSE5*, indicating a genetic interaction between *OsPUB3* and *GSE5*. We also analyzed the relationship between *OsPUB3* and *GS3*. Interactions between the two genes were not evident, and their effects were additive with each other (data not shown).

*OsPUB3* encodes a U-box/ARM repeat protein possessing E3 ubiquitin ligase activity. The U-box domain containing approximately 70 amino acid residues is a modified RING-finger domain, which could recruit E2 proteins for ubiquitination of pre-mRNA splicing complexes and unfolded proteins associated with the proline-isomerase chaperone, respectively (Aravind et al. 2000). The ARM repeat containing approximately 40 amino acid residues is a short leucine-rich protein-interacting domain, which serve to recognize and recruit the substrate (Zeng et al. 2008). So far, genetic effects of the other regions on U-box/ARM E3 ligase have not been reported. In this study, six SNPs were found in the coding region of *OsPUB3*. Four of them caused amino acid changes. Compared with Nipponbare (Hap2), SNP1 (C97T), SNP3 (C755T), SNP4 (A830G) and SNP5 (A1546T) led to amino acids change from Pro to Ser (P33S), Thr to Met (T252M), Asp to Gly (D277G), and Thr to Ser (T516S), respectively. The SNP4 is located in the U-box domain and the other three SNPs are not located in either the U-box domain or ARM (Additional file 1: Figure S5). This SNP is monomorphic among the three major haplotypes of *OsPUB3*, Hap1, Hap2 and Hap3, so is SNP3 (Additional file 2: Table S2). Thus, trait differences among these haplotypes may be ascribed to the other two SNPs. At SNP1, Hap1 and Hap3 carried the same allele but Hap2 had the other allele. Compared with Hap1 and Hap3, Hap2 was lower in GL, higher in GW and lower in RLW (Additional file 1: Figure S3). For TGW, Hap2 has a higher value compared with Hap1, but did not differ significantly from Hap3. These indicate that SNP1 may be responsible for grain shape rather than for grain size. At SNP5, Hap2 and Hap3 carried the same allele but Hap1 had the other allele. Compared with Hap2 and Hap3, Hap1 was different on all the three grain-size traits analyzed, suggesting that SNP5 may have an important role in controlling grain size.

As shown in Fig. 2, decreases of GW and TGW in the two KO mutants led to decreases in grain yield per plot, although the change was statistically non-significant. On the other hand, the two KO mutants showed higher HRR that is an indication of better milling quality. These results are in agreement with the understanding that rice grain yield and quality traits are often negatively correlated with one another (Sakamoto et al. 2008). However, with the cloning of more and more genes regulating component traits of rice grain yield and quality, it has been shown that the negative correlation between yield and quality can be overcome by combining complementary genes. For instance, combining the *GW7*^TFA^ allele with *gw8*^Basmati^ allele (Wang et al. 2015a), or *OsMADS1*^lgy3^ allele with *dep1-1* and *gs3* alleles could simultaneously improve rice grain yield and quality (Liu et al. 2018). In addition, creation of new allelic variations through genome editing also provided a novel strategy to solve negative correlations between different traits. For *IPA1* as a rice gene increasing grains per panicle but decreasing tillers (Jiao et al. 2010), a novel allele *ipa1-pro10* simultaneously increasing grains per panicle and tillers was created using a tiling-deletion-based CRISPR-Cas9 screen (Song et al. 2022). Among the three major haplotypes of *OsPUB3*, Hap3 has the intermediate grain width but largest grain weight and grain length (Additional file 1: Figure S3). Thus, a favorable slender grain shape may be accompanied by a higher grain weight. Therefore, this haplotype exhibited a potential in rice breeding for simultaneously improving rice grain yield and quality.

It is often observed that genes controlling the same trait are closely linked. Cloned genes showing major effects on rice grain weight and size have provided clear examples. Two gene clusters for GL on chromosome 3, *OsLG3* and *OsLG3b*/*qLGY3*, and *GS3* and *SG3*, are located in a 1.7-Mb region on the short arm and a 0.2-Mb region in the pericentromeric region, respectively (Fan et al. 2006; Yu et al. 2017, 2018; Liu et al. 2018; Li et al. 2020). *TGW6*, *GW6a* and *GL6* for grain weight are in a 1.6-Mb region on the long arm of chromosome 6 (Ishimaru et al. 2013; Song et al. 2015; Wang et al. 2019a). In our previous studies, four minor-effect QTLs for grain size and its component traits, including *qTGW1.2a*, *qTGW1.2b*, *qGS1-35.2* and *qGW1-35.5*, were dissected in a 4.5-Mb region on the long arm of chromosome 1 (Wang et al. 2015c; Dong et al. 2018; Wang et al. 2019b). We reported the cloning of *qTGW1.2b* in 2021(Chan et al. 2021) and that of *qGS1-35.2* here. Recently, Shang et al. (2022) reported that *LOC_Os01g57250* encoding an unknown expressed protein is the likely causal gene underlying *qTGW1.2a*. These results indicate that minor-effect genes controlling the same trait are also generally close linked.

## Materials and Methods

### Plant Materials

Two sets of rice materials were used in this study. One contained *OsPUB3* KO mutants, the other contained *OsPUB3* transgenic complementation plants.

*OsPUB3* KO mutants in the background of ZH161 were produced by using the CRISPR/Cas9 system. The recipient ZH161 has the same genomic sequence at *OsPUB3* locus as MY46 which was the male parent of the mapping population for *OsPUB3*. Two independent T_0_ homozygous transgenic plants, KO-1 and KO-2, were obtained. Both the mutants had a 1-bp insertion at + 91 in the coding region (Fig. 1A). The T_1_ and T_2_ knock-out populations derived from the two homozygous mutants were used to test the genetic effects of *OsPUB3* on grain size and other yield and quality traits.

*OsPUB3* transgenic complementation plants were produced in the background of a Cas9-free progeny of the mutant KO-1. Four independent T_0_ transgenic-positive plants were obtained. Each T_0_ plant was selfed to produce a T_1_ population segregating at the target gene locus. In each T_1_ population, individual plants were genotyped and measured for grain size traits. The four T_1_ complementation populations were used to verify that *OsPUB3* is the cause gene for *qGS1-35.2*.

### Field Experiment and Phenotyping

All the rice materials were grown in the paddy field of China National Rice Research Institute located at Hangzhou, Zhejiang Province, China. The plants were grown at a spacing of 16.7 cm between plants and 26.7 cm between rows. Field management followed local agricultural practice.

For the KO experiment, T_1_ and T_2_ plants of the two KO mutants were tested with the recipient ZH161 and a transgenic-negative control. The T_1_ lines were grown in 2019, in which 20 plants were included for each of the four genotypes. At maturity, each plant was harvested and sun-dried. Three traits were measured, including TGW, GL and GW. Two samples of 100 fully filled grains were randomly selected for measurement using an electronic image analysis system (Model SC-G, Wanshen Ltd., Hangzhou, China). The T_2_ lines were grown in 2021. Three replications of 36 plants were included for each of the four genotypes. The 36 plants were planted as three rows of 12 plants. HD was recorded using 12 plants in the second row and averaged. At maturity, middle five plants in the second row were harvested in bulk and measured for NP and NGP. Other plants of each plot were harvested, weighted and added to measure grain yield per plot. Fully filled grains were randomly selected and measured for TGW, GL and GW following the method reported by Zhang et al. (2016). Three traits of rice milling quality traits, BRR, MRR and HRR, were measured following the national standard NY/T 83-2017.

For the complementation experiment, four T_1_ populations were grown in 2021, in which each population included 108 plants. At maturity, the three grain size traits, TGW, GL and GW, were measured based on a single-plant basis.

### Construction of the Knock-Out and Complementary Vectors

For the KO experiment, the target site which was designed applying the web-based tool CRISPR-GE was located at + 74 to + 93 in exon 1 (Fig. 1A). Oligonucleotide Cri-PUB3 (Additional file 2: Table S4) was ligated into the CRISPR/Cas9 expression vector BGK03 following the manufacturer’s instructions (Biogle Co., Ltd., Hangzhou, China). The BGK03 vector comprised a rice U6 promoter for activating the target site sequence, a Cas9 gene driven by the maize ubiquitin promoter and a hygromycin marker gene driven by Cauliflower mosaic virus 35S promoter. The expression vector was introduced into ZH161 using Agrobacterium tumefaciens-mediated transformation.

For the complementation experiment, a DNA fragment of 6176 bp containing the 2879-bp promoter, the 2043-bp coding region, and the 1254-bp termination site was amplified from the genomic DNA of MY46 and cloned into binary vector pCAMBIA1300. The pCAMBIA1300 vector comprised a kanamycin resistance gene for bacterial selection and a hygromycin resistance gene for plant selection. The hygromycin resistance gene was driven by a double-enhancer version of the Cauliflower mosaic virus 35S promoter. The target gene expression was driven by its own promoter. The expression vector was introduced into Tc3-6, a Cas9-free progeny of KO-1 using Agrobacterium tumefaciens-mediated transformation.

### Detection of Transgenic Plants

Genomic DNA of the T_0_ plants was extracted from young leaves using the DNeasy Plant Mini Kit (Qiagen, Hilden, Germany). For the KO experiment, T_0_ transgenic plants were identified by using the hygromycin gene marker Hyg. The *OsPUB3* gene fragment surrounding the target region was amplified from the Hyg-positive plant using the primer pairs Seq-PUB3. The PCR fragments were directly sequenced by Sanger method and decoded using the web-based tool DSDecodeM (http://skl.scau.edu.cn/dsdecode). Two independent T_0_ homozygous mutants were obtained. Each T_0_ mutant was selfed to produce a T_1_ population. Eight plants from each T_1_ population were used to verify the sequence variation by sequencing. For the complementation experiment, T_0_ transgenic plants were also identified by using the hygromycin gene marker Hyg. Four independent Hyg-positive plants were obtained. Each of them was selfed to produce a T_1_ population. In each resultant T_1_ population, DNA of each plant was extracted from young leaves using a mini-preparation method (Zheng et al. 1995). Individual plants of each T_1_ population were genotyped using the primer pairs Seq-PUB3 that was used to detect the sequence variation of the target region for knockout mutants. Primers are listed in Additional file 2: Table S4.

### Data Analysis

For the KO experiment, the Duncan’s multiple range test was employed to test the phenotypic differences among the KO mutants, transgenic-negative control and recipient. Any two groups having a common letter, are not significantly different at a 0.05 level of significance. Instead, any two groups with different letters are significantly different at a 0.05 level of significance. For the complementation experiment, the Student’s *t*-test was employed to test the phenotypic differences (*p* < 0.05) between transgenic negative and positive segregants.

### Microscopy Observation

CK and KO-1 were used for observation of outer glume epidermal cell. Young spikelet hulls were fixed with 2.5% glutaraldehyde for 24 h and then dehydrated in a graded series of ethanol (30%, 50%, 75%, 95%, 100%, 100% and 100%). The dehydrated sample were coated with gold–palladium using ion sputter (Model E-1010, Hitachi, Japan) and observed using scanning electron microscope (Model TM-1000, Hitachi, Japan). Cell length and width of the outer glumes were measured, then cell number in the transverse and longitudinal direction were calculated. Twenty glumes were used for CK and KO-1 respectively. The Student’s *t*-test was employed to test the differences in cell length, width and numbers between CK and KO-1 (*p* < 0.05).

### Haplotype Analysis of *OsPUB3*

Haplotype analysis was firstly analyzed by using data documented in RiceVarMap V2.0 database (http://ricevarmap.ncpgr.cn). Data on the genomic variations in the *OsPUB3* coding region of all rice accessions in the database was obtained by using the “Search for Variation by Gene” function. Data on the *OsPUB3* haplotypes were downloaded from the website.

The allelic variation of *OsPUB3* was further analyzed using 257 *indica* rice cultivars. Total DNA was extracted from a single plant of each germplasm using the method of Zheng et al. (1995). The fragment covering the *OsPUB3* gene was amplified using the primer pairs S3900. The PCR fragments were directly sequenced by Sanger method. Sequences were aligned by Clustal W program (University College Dublin, Dublin, Ireland) and analyzed with MEGA 6.0 software (Mega Ltd., Auckland, New Zealand). For *GSE5*, one primer pairs, GSE5-Del, was designed according to the sequence variation in the promoter of *GSE5* (Duan et al. 2017).

## Conclusion

Decreased grain weight and size owing to gene knock-out is accordance with increased grain weight and size due to genetic complementation, indicating that *OsPUB3* is a gene positively regulating grain weight and size in rice. Genetic interaction between *OsPUB3* and *GSE5* was detected. The *OsPUB3* showed larger effects on grain weight and size in the non-functional *gse5* background but not in the functional *GSE5* background. Cloning of *OsPUB3* provides a new gene resource for investigating the regulation of grain size and its utilization in rice breeding.

## Supplementary Information

**Additional file 1**.** Figure S1**. Characterization of the cells in the outer glume epidermal of CK and KO-1 for OsPUB3.** A** Comparison of cell length in the outer glume epidermal.** B** Comparison of cell width in the outer glume epidermal.** C** Comparison of cell numbers in the longitudinal direction.** D** Comparison of cell numbers in the transverse direction.** E** Scanning electron microscopic images of the cells. Scale bars=100 μm. Values are given as the mean ± SD (*n* = 20). Differences between CK and KO-1 were tested by student’s t-test. *, p < 0.05; ns: not significant.** Figure S2**. The effects of OsPUB3 knock-out on grain width, grain length and head rice recovery.** A** Comparisons of grain width among the knock-out mutants and the two controls.** B** Comparisons of grain length among the knock-out mutants and the two controls.** C** Comparisons of head rice recovery among the knock-out mutants and the two controls. Scale bars = 10 mm.** Figure S3**. Phenotypic differences among three haplotypes of OsPUB3.** A** 1,000-grain weight.** B** Grain length.** C** Grain width.** D** Ratio of grain length to width. Values are given as the mean ± SD (*n* = 338 for Hap1,* n* = 122 for Hap2,* n* = 50 for Hap3). Values with different letters are significantly different at p < 0.05 based on the Duncan’s multiple range test.** Figure S4**. Phenotypic differences between ZS97-type and MY46-type of OsPUB3.** A**-**C** Three traits measured in Lingshui trial. D-F Three traits measured in Hangzhou trial. Values are given as the mean ± SD (* n* = 19 for ZS97-type;* n* = 230 for MY46-type). *, p < 0.05; **, p < 0.01; ns: not significant.** Figure S5**. Protein variations of the eight haplotypes. Difference in amino acid is shown in green.

**Additional file 2**.** Table S1**. Phenotypic change due to OsPUB3 knockout.** Table S2**. Haplotypes of OsPUB3 in rice germplasms.** Table S3**. Rice germplasms used in this study.** Table S4**. Primers used in this study.

## Data Availability

All data supporting the findings of this study are available from the corresponding author on reasonable request.

## Abbreviations

BRR: Brown rice recovery
HD: Heading date
HRR: Head rice recovery
GL: Grain length
GW: Grain width
GY: Grain yield per plot
MRR: Milled rice recovery
MY46: Milyang 46
NGP: Number of grains per panicle
NP: Number of panicles per plant
QTL: Quantitative trait locus
TGW: 1000-Grain weight
ZH161: Zhonghui 161
ZS97: Zhenshan 97

## References

[CR1] Aravind L, Koonin EV (2000). The U box is a modified RING finger-a common domain in ubiquitination. Curr Biol.

[CR2] Bao J (2019) Rice chemistry and technology. In: Bao J (ed) Rice milling quality, 4th ed. United Kingdom, pp 339–369

[CR3] Chan AN, Wang LL, Zhu YJ, Fan YY, Zhuang JY, Zhang ZH (2021). Identification through fine mapping and verification using CRISPR/Cas9-targeted mutagenesis for a minor QTL controlling grain weight in rice. Theor Appl Genet.

[CR4] Che R, Tong H, Shi B, Liu Y, Fang S, Liu D, Xiao Y, Hu B, Liu L, Wang H, Zhao M, Chu C (2016). Control of grain size and rice yield by GL2-mediated brassinosteroid responses. Nat Plants.

[CR5] Dong Q, Zhang ZH, Wang LL, Zhu YJ, Fan YY, Mou TM, Ma LY, Zhuang JY (2018). Dissection and fine-mapping of two QTL for grain size linked in a 460-kb region on chromosome 1 of rice. Rice.

[CR6] Dong NQ, Sun Y, Guo T, Shi CL, Zhang YM, Kan Y, Xiang YH, Zhang H, Yang YB, Li YC, Zhao HY, Yu HX, Lu ZQ, Wang Y, Ye WW, Shan JX, Lin HX (2020). UDP-glucosyltransferase regulates grain size and abiotic stress tolerance associated with metabolic flux redirection in rice. Nat Commun.

[CR7] Duan P, Xu J, Zeng D, Zhang B, Geng M, Zhang G, Huang K, Huang L, Xu R, Ge S, Qian Q, Li Y (2017). Natural variation in the promoter of *GSE5* contributes to grain size diversity in rice. Mol Plant.

[CR8] Fan C, Xing Y, Mao H, Lu T, Han B, Xu C, Li X, Zhang Q (2006). *GS3*, a major QTL for grain length and weight and minor QTL for grain width and thickness in rice, encodes a putative transmembrane protein. Theor Appl Genet.

[CR9] Hu J, Wang Y, Fang Y, Zeng L, Xu J, Yu H, Shi Z, Pan J, Zhang D, Kang S, Zhu L, Dong G, Guo L, Zeng D, Zhang G, Xie L, Xiong G, Li J, Qian Q (2015). A rare allele of *GS2* enhances grain size and grain yield in Rice. Mol Plant.

[CR10] Hu Z, Lu SJ, Wang MJ, He H, Sun L, Wang H, Liu X, Jiang L, Sun JL, Xin X, Kong W, Chu C, Xue HW, Yang J, Luo X, Liu JX (2018). A novel QTL *qTGW3* encodes the GSK3/SHAGGY-like kinase OsGSK5/OsSK41 that interacts with OsARF4 to negatively regulate grain size and weight in rice. Mol Plant.

[CR11] Ishimaru K, Hirotsu N, Madoka Y, Murakami N, Hara N, Onodera H, Kashiwagi T, Ujiie K, Shimizu B, Onishi A, Miyagawa H, Katoh E (2013). Loss of function of the IAA-glucose hydrolase gene *TGW6* enhances rice grain weight and increases yield. Nat Genet.

[CR12] Jiao Y, Wang Y, Xue D, Wang J, Yan M, Liu G, Dong G, Zeng D, Lu Z, Zhu X, Qian Q, Li J (2010). Regulation of OsSPL14 by OsmiR156 defines ideal plant architecture in rice. Nat Genet.

[CR13] Li Y, Fan C, Xing Y, Jiang Y, Luo L, Sun L, Shao D, Xu C, Li X, Xiao J, He Y, Zhang Q (2011). Natural variation in *GS5* plays an important role in regulating grain size and yield in rice. Nat Genet.

[CR14] Li S, Gao F, Xie K, Zeng X, Cao Y, Zeng J, He Z, Ren Y, Li W, Deng Q, Wang S, Zheng A, Zhu J, Liu H, Wang L, Li P (2016). The OsmiR396c-OsGRF4-OsGIF1 regulatory module determines grain size and yield in rice. Plant Biotechnol J.

[CR15] Li Q, Lu L, Liu H, Bai X, Zhou X, Wu B, Yuan M, Yang L, Xing Y (2020). A minor QTL, *SG3*, encoding an R2R3-MYB protein, negatively controls grain length in rice. Theor Appl Genet.

[CR16] Liu J, Chen J, Zheng X, Wu F, Lin Q, Heng Y, Tian P, Cheng Z, Yu X, Zhou K, Zhang X, Guo X, Wang J, Wang H, Wan J (2017). *GW5* acts in the brassinosteroid signalling pathway to regulate grain width and weight in rice. Nat Plants.

[CR17] Liu Q, Han R, Wu K, Zhang J, Ye Y, Wang S, Chen J, Pan Y, Li Q, Xu X, Zhou J, Tao D, Wu Y, Fu X (2018). G-protein βγ subunits determine grain size through interaction with MADS-domain transcription factors in rice. Nat Commun.

[CR18] Mao X, Yu C, Li L, Wang M, Yang L, Zhang Y, Zhang Y, Wang J, Li C, Reynolds MP, Jing R (2022). How many faces does the plant U-Box E3 ligase have?. Int J Mol Sci.

[CR19] Qi P, Lin YS, Song XJ, Shen JB, Huang W, Shan JX, Zhu MZ, Jiang L, Gao JP, Lin HX (2012). The novel quantitative trait locus GL3.1 controls rice grain size and yield by regulating Cyclin-T1;3. Cell Res.

[CR20] Qiao J, Jiang H, Lin Y, Shang L, Wang M, Li D, Fu X, Geisler M, Qi Y, Gao Z, Qian Q (2021). A novel *miR167a-OsARF6-OsAUX3* module regulates grain length and weight in rice. Mol Plant.

[CR21] Ruan B, Shang L, Zhang B, Hu J, Wang Y, Lin H, Zhang A, Liu C, Peng Y, Zhu L, Ren D, Shen L, Dong G, Zhang G, Zeng D, Guo L, Qian Q, Gao Z (2020). Natural variation in the promoter of *TGW2* determines grain width and weight in rice. New Phytol.

[CR22] Sakamoto T, Matsuoka M (2008). Identifying and exploiting grain yield genes in rice. Curr Opin Plant Biol.

[CR23] Shang L, Li X, He H, Yuan Q, Song Y, Wei Z, Lin H, Hu M, Zhao F, Zhang C, Li Y, Gao H, Wang T, Liu X, Zhang H, Zhang Y, Cao S, Yu X, Zhang B, Hu Z, Wang H, Lv Y, Wang Y, Ma J, Wang Q, Lu H, Wu Z, Liu S, Sun Z, Zhang H, Guo L, Li Z, Zhou Y, Li J, Zhu Z, Xiong G, Ruan J, Qian Q (2022). A super pan-genomic landscape of rice. Cell Res.

[CR24] Shi CL, Dong NQ, Guo T, Ye WW, Shan JX, Lin HX (2020). A quantitative trait locus *GW6* controls rice grain size and yield through the gibberellin pathway. Plant J.

[CR25] Si L, Chen J, Huang X, Gong H, Luo J, Hou Q, Zhou T, Lu T, Zhu J, Shangguan Y, Chen E, Gong D, Lu Y, Weng Q, Wang Y, Zhan Q, Liu K, Wei X, An K, An G, Han B (2016). *OsSPL13* controls grain size in cultivated rice. Nat Genet.

[CR26] Song XJ, Huang W, Shi M, Zhu MZ, Lin HX (2007). A QTL for rice grain width and weight encodes a previously unknown RING-type E3 ubiquitin ligase. Nat Genet.

[CR27] Song XJ, Kuroha T, Ayano M, Furuta T, Nagai K, Komeda N, Segami S, Miura K, Ogawa D, Kamura T, Suzuki T, Higashiyama T, Yamasaki M, Mori H, Inukai Y, Wu J, Kitano H, Sakakibara H, JacobsenAshikari SM (2015). Rare allele of a previously unidentified histone H4 acetyltransferase enhances grain weight, yield, and plant biomass in rice. Proc Natl Acad Sci USA.

[CR28] Song X, Meng X, Guo H, Cheng Q, Jing Y, Chen M, Liu G, Wang B, Wang Y, Li J, Yu H (2022). Targeting a gene regulatory element enhances rice grain yield by decoupling panicle number and size. Nat Biotechnol.

[CR29] Wang S, Wu K, Yuan Q, Liu X, Liu Z, Lin X, Zeng R, Zhu H, Dong G, Qian Q, Zhang G, Fu X (2012). Control of grain size, shape and quality by *OsSPL16* in rice. Nat Genet.

[CR30] Wang S, Li S, Liu Q, Wu K, Zhang J, Wang S, Wang Y, Chen X, Zhang Y, Gao C, Wang F, Huang H, Fu X (2015). The *OsSPL16-GW7* regulatory module determines grain shape and simultaneously improves rice yield and grain quality. Nat Genet.

[CR31] Wang Y, Xiong G, Hu J, Jiang L, Yu H, Xu J, Fang Y, Zeng L, Xu E, Xu J, Ye W, Meng X, Liu R, Chen H, Jing Y, Wang Y, Zhu X, Qian Q (2015). Copy number variation at the *GL7* locus contributes to grain size diversity in rice. Nat Genet.

[CR32] Wang LL, Chen YY, Guo L, Zhang HW, Fan YY, Zhuang JY (2015). Dissection of *qTGW1.2* to three QTLs for grain weight and grain size in rice (*Oryza sativa* L.). Euphytica.

[CR33] Wang A, Hou Q, Si L, Huang X, Luo J, Lu D, Zhu J, Shangguan Y, Miao J, Xie Y, Wang Y, Zhao Q, Feng Q, Zhou C, Li Y, Fan D, Lu Y, Tian Q, Wang Z, Han B (2019). The PLATZ transcription factor GL6 affects grain length and number in rice. Plant Physiol.

[CR34] Wang W, Wang L, Zhu Y, Fan Y, Zhuang J (2019). Fine mapping of qTGW1.2a, a quantitative trait locus for 1000-grain weight in rice. Rice Sci.

[CR35] Weng J, Gu S, Wan X, Gao H, Guo T, Su N, Lei C, Zhang X, Cheng Z, Guo X, Wang J, Jiang L, Zhai H, Wan J (2008). Isolation and initial characterization of *GW5*, a major QTL associated with rice grain width and weight. Cell Res.

[CR36] Xie L, Tang S, Chen N, Luo J, Jiao G, Shao G, Wei X, Hu P (2013). Rice grain morphological characteristics correlate with grain weight and milling quality. Cereal Chem.

[CR37] Yu J, Xiong H, Zhu X, Zhang H, Li H, Miao J, Wang W, Tang Z, Zhang Z, Yao G, Zhang Q, Pan Y, Wang X, Rashid MAR, Li J, Gao Y, Li Z, Fu X, Li Z (2017). *OsLG3* contributing to rice grain length and yield was mined by Ho-LAMap. BMC Biol.

[CR38] Yu J, Miao J, Zhang Z, Xiong H, Zhu X, Sun X, Pan Y, Liang Y, Zhang Q, Rashid MAR, Li J, Zhang H, Li Z (2018). Alternative splicing of *OsLG3b* controls grain length and yield in *japonica* rice. Plant Biotechnol J.

[CR39] Zeng LR, Park CH, Venu RC, Gough J, Wang GL (2008). Classification, expression pattern, and E3 ligase activity assay of rice U-box-containing proteins. Mol Plant.

[CR40] Zhan P, Wei X, Xiao Z, Wang X, Ma S, Lin S, Li F, Bu S, Liu Z, Zhu H, Liu G, Zhang G, Wang S (2021). *GW10*, a member of P450 subfamily regulates grain size and grain number in rice. Theor Appl Genet.

[CR41] Zhan P, Ma S, Xiao Z, Li F, Wei X, Lin S, Wang X, Ji Z, Fu Y, Pan J, Zhou M, Liu Y, Chang Z, Li L, Bu S, Liu Z, Zhu H, Liu G, Zhang G, Wang S (2022). Natural variations in *grain length 10 (GL10)* regulate rice grain size. J Genet Genom.

[CR42] Zhang HW, Fan YY, Zhu YJ, Chen JY, Yu SB, Zhuang JY (2016). Dissection of the qTGW1.1 region into two tightly-linked minor QTLs having stable effects for grain weight in rice. BMC Genet.

[CR43] Zhang YM, Yu HX, Ye WW, Shan JX, Dong NQ, Guo T, Kan Y, Xiang YH, Zhang H, Yang YB, Li YC, Zhao HY, Lu ZQ, Guo SQ, Lei JJ, Liao B, Mu XR, Cao YJ, Yu JJ, Lin HX (2021). A rice QTL GS3.1 regulates grain size through metabolic-flux distribution between flavonoid and lignin metabolons without affecting stress tolerance. Commun Biol.

[CR44] Zhao H, Yao W, Ouyang Y, Yang W, Wang G, Lian X, Xing Y, Chen L, Xie W (2015). RiceVarMap: a comprehensive database of rice genomic variations. Nucleic Acids Res.

[CR45] Zhao DS, Li QF, Zhang CQ, Zhang C, Yang QQ, Pan LX, Ren XY, Lu J, Gu MH, Liu QQ (2018). *GS9* acts as a transcriptional activator to regulate rice grain shape and appearance quality. Nat Commun.

[CR46] Zhao H, Li J, Yang L, Qin G, Xia C, Xu X, Su Y, Liu Y, Ming L, Chen LL, Xiong L, Xie W (2021). An inferred functional impact map of genetic variants in rice. Mol Plant.

[CR47] Zheng KL, Huang N, Bennett J, Khush GS (1995) PCR-based marker-assisted selection in rice breeding: IRRI Discussion Paper Series No. 12. Los Banos: International Rice Research Institute

[CR48] Zuo J, Li J (2014). Molecular genetic dissection of quantitative trait loci regulating rice grain size. Annu Rev Genet.

[CR49] Zuo ZW, Zhang ZH, Huang DR, Fan YY, Yu SB, Zhuang JY, Zhu YJ (2022). Control of thousand-grain weight by *OsMADS56* in rice. Int J Mol Sci.

