# Supplementary material for: Control of Grain Weight and Size in Rice (Oryza sativa L.) by OsPUB3 Encoding a U-Box E3 Ubiquitin Ligase
Source: Rice (N Y). 2022 Nov 23;15:58. doi: 10.1186/s12284-022-00604-1 (PMC9684364; doi:10.1186/s12284-022-00604-1)
Supplement: Supplementary file 1 — Additional file 1. Figure S1. Characterization of the cells in the outer glume epidermal of CK and KO-1 for OsPUB3. A Comparison of cell length in the outer glume epidermal. B Comparison of cell width in the outer glume epidermal. C Comparison of cell numbers in the longitudinal direction. D Comparison of cell numbers in the transverse direction. E Scanning electron microscopic images of the cells. Scale bars=100 μm. Values are given as the mean ± SD (n = 20). Differences between CK and KO-1 were tested by student’s t-test. *, p < 0.05; ns: not significant. Figure S2. The effects of OsPUB3 knock-out on grain width, grain length and head rice recovery. A Comparisons of grain width among the knock-out mutants and the two controls. B Comparisons of grain length among the knock-out mutants and the two controls. C Comparisons of head rice recovery among the knock-out mutants and the two controls. Scale bars = 10 mm. Figure S3. Phenotypic differences among three haplotypes of OsPUB3. A 1,000-grain weight. B Grain length. C Grain width. D Ratio of grain length to width. Values are given as the mean ± SD (n = 338 for Hap1, n = 122 for Hap2, n = 50 for Hap3). Values with different letters are significantly different at p < 0.05 based on the Duncan’s multiple range test. Figure S4. Phenotypic differences between ZS97-type and MY46-type of OsPUB3. A-C Three traits measured in Lingshui trial. D-F Three traits measured in Hangzhou trial. Values are given as the mean ± SD ( n = 19 for ZS97-type; n = 230 for MY46-type). *, p < 0.05; **, p < 0.01; ns: not significant. Figure S5. Protein variations of the eight haplotypes. Difference in amino acid is shown in green. [file 12284_2022_604_MOESM1_ESM.docx]

**Figure S1.** Characterization of the cells in the outer glume epidermal of CK and KO-1 for *OsPUB3*. **A** Comparion of cell length in the outer glume epidermal. **B** Comparion of cell width in the outer glume epidermal. **C** Comparion of cell numbers in the longitudinal direction. **D** Comparion of cell numbers in the transverse direction. **E** Scanning electron microscopic images of the cells. Scale bars=100 μm. Values are given as the mean ± SD (*n*=20). Differences between CK and KO-1 were tested by student’s *t*-test. *, *p* < 0.05; ns: not significant.

**Figure S2.** The effects of *OsPUB3* knock-out on grain width, grain length and head rice recovery. **A** Comparisons of grain width among the knock-out mutants and the two controls. **B** Comparisons of grain length among the knock-out mutants and the two controls. **C** Comparisons of head rice recovery among the knock-out mutants and the two controls. Scalebars = 10 mm.


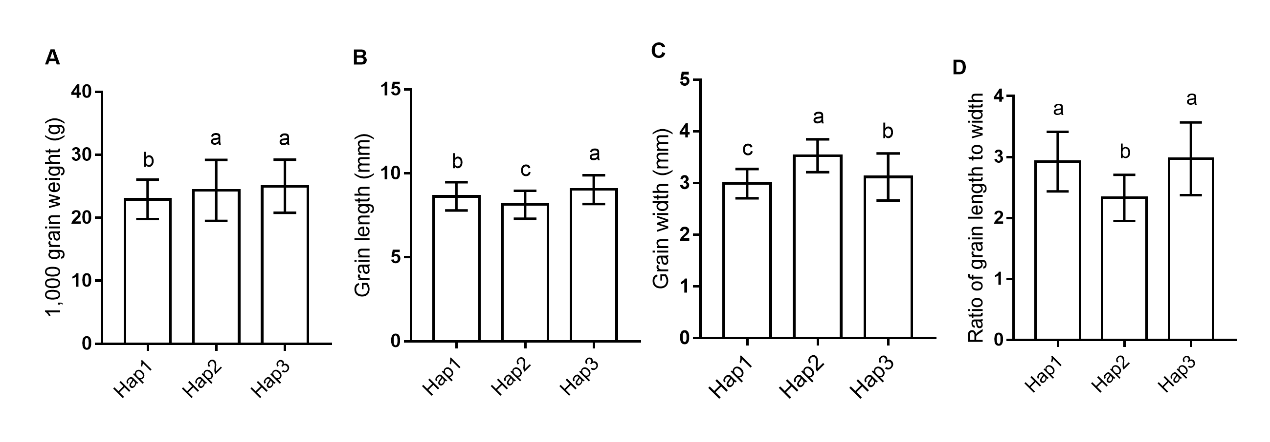


**Figure S3.** Phenotypic differences among three haplotypes of *OsPUB3*. **A** 1,000-grain weight. **B** Grain length. **C** Grain width. **D** Ratio of grain length to width. Values are given as the mean ± SD (*n* = 338 for Hap1, *n* = 122 for Hap2, *n* = 50 for Hap3). Values with different letters are significantly different at *p* < 0.05 based on the Duncan’s multiple range test.


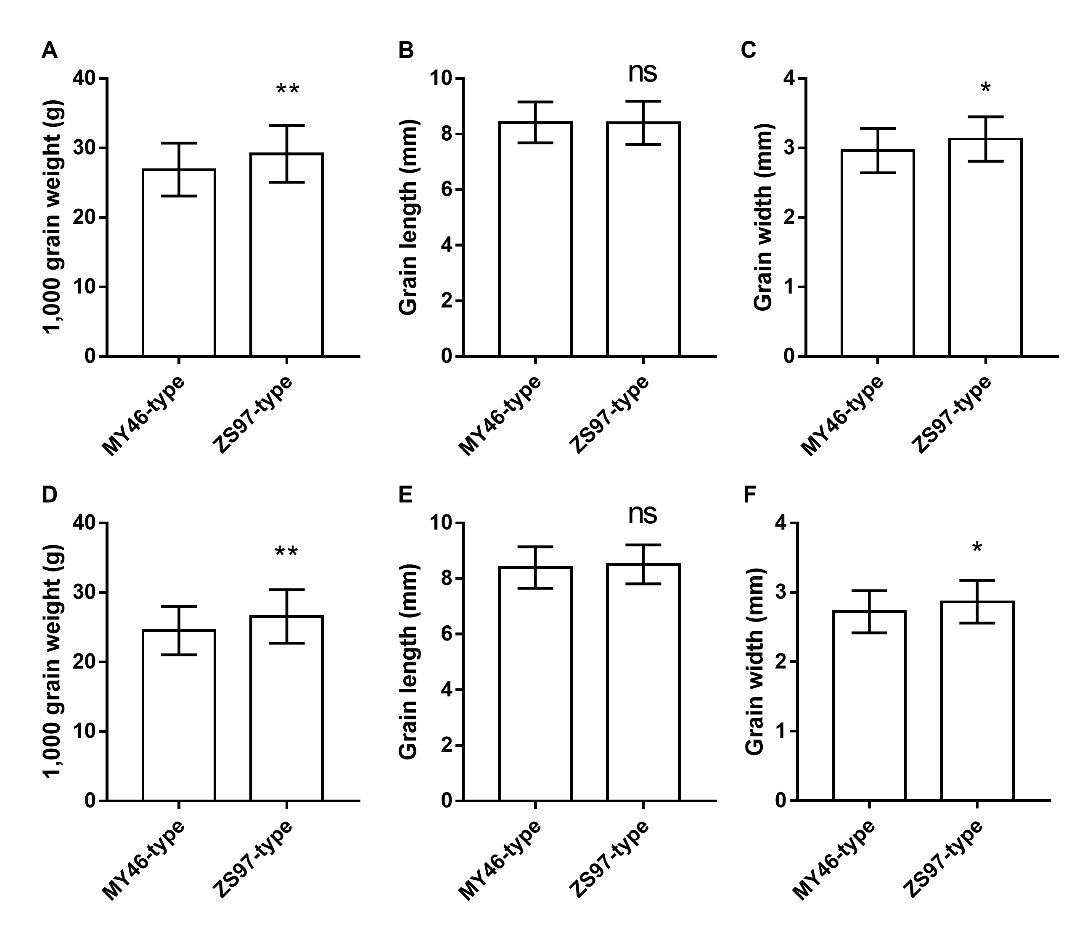


**Figure S4.** Phenotypic differences between ZS97-type and MY46-type of *OsPUB3*. **A-C** Three traits measured in Lingshui trial. **D-F** Three traits measured in Hangzhou trial. Values are given as the mean ± SD (*n*=19 for ZS97-type; *n*=230 for MY46-type). *, *p* < 0.05; **, *p* < 0.01; ns: not significant.

**Figure S5.** Protein variations of the eight haplotypes. Difference in amino acid is shown in green.
