# Supplementary material for: Control of Grain Weight and Size in Rice (Oryza sativa L.) by OsPUB3 Encoding a U-Box E3 Ubiquitin Ligase
Source: Rice (N Y). 2022 Nov 23;15:58. doi: 10.1186/s12284-022-00604-1 (PMC9684364; doi:10.1186/s12284-022-00604-1)
Supplement: Supplementary file 2 — Additional file 2. Table S1. Phenotypic change due to OsPUB3 knockout. Table S2. Haplotypes of OsPUB3 in rice germplasms. Table S3. Rice germplasms used in this study. Table S4. Primers used in this study. [file 12284_2022_604_MOESM2_ESM.docx]

**Table S**1. Phenotypic change due to *OsPUB3* knock-out.

| Trial ^a^ | Trait ^b^ | Line | Mean ± SD ^c^ | ±RP ^d^ | ±RP% ^e^ | ±CK ^f^ | ±CK% ^g^ |
| --- | --- | --- | --- | --- | --- | --- | --- |
| T1 | TGW | ZH161 | 19.04 ± 0.45a |  |  |  |  |
|  |  | CK | 19.23 ± 0.48a |  |  |  |  |
|  |  | KO-1 | 17.50 ± 0.51c | -1.54 | -8.1 | -1.73 | -9.0 |
|  |  | KO-2 | 18.47 ± 0.41b | -0.57 | -3.0 | -0.76 | -3.9 |
|  | GL | ZH161 | 7.656 ± 0.065c |  |  |  |  |
|  |  | CK | 7.863 ± 0.067b |  |  |  |  |
|  |  | KO-1 | 7.598 ± 0.113d | -0.057 | -0.7 | -0.265 | -3.4 |
|  |  | KO-2 | 7.933 ± 0.084a | 0.277 | 3.6 | 0.070 | 0.9 |
|  | GW | ZH161 | 2.452 ± 0.030a |  |  |  |  |
|  |  | CK | 2.451 ± 0.042a |  |  |  |  |
|  |  | KO-1 | 2.369 ± 0.040b | -0.084 | -3.4 | -0.083 | -3.4 |
|  |  | KO-2 | 2.358 ± 0.034b | -0.094 | -3.8 | -0.093 | -3.8 |
| T2 | TGW | ZH161 | 20.30 ± 0.27a |  |  |  |  |
|  |  | CK | 20.72 ± 0.04a |  |  |  |  |
|  |  | KO-1 | 18.62 ± 0.32c | -1.68 | -8.3 | -2.09 | -10.1 |
|  |  | KO-2 | 19.59 ± 0.24b | -0.71 | -3.5 | -1.13 | -5.4 |
|  | GL | ZH161 | 7.959 ± 0.091b |  |  |  |  |
|  |  | CK | 8.146 ± 0.030a |  |  |  |  |
|  |  | KO-1 | 7.712 ± 0.084c | -0.246 | -3.1 | -0.433 | -5.3 |
|  |  | KO-2 | 8.140 ± 0.016a | 0.182 | 2.3 | -0.006 | -0.1 |
|  | GW | ZH161 | 2.605 ± 0.009a |  |  |  |  |
|  |  | CK | 2.572 ± 0.011a |  |  |  |  |
|  |  | KO-1 | 2.512 ± 0.014b | -0.093 | -3.6 | -0.059 | -2.3 |
|  |  | KO-2 | 2.477 ± 0.040b | -0.128 | -4.9 | -0.095 | -3.7 |
|  | NP | ZH161 | 12.8 ± 3.4a |  |  |  |  |
|  |  | CK | 13.7 ± 1.1a |  |  |  |  |
|  |  | KO-1 | 12.9 ± 1.5a | 0.1 | 0.5 | -0.9 | -6.3 |
|  |  | KO-2 | 14.3 ± 0.5a | 1.5 | 11.5 | 0.5 | 3.9 |
|  | NGP | ZH161 | 129.6 ± 9.5ab |  |  |  |  |
|  |  | CK | 115.5 ± 8.1b |  |  |  |  |
|  |  | KO-1 | 138.4 ± 3.6a | 8.8 | 6.8 | 22.9 | 19.8 |
|  |  | KO-2 | 123.8 ± 6.7b | -5.8 | -4.5 | 8.3 | 7.2 |
|  | GY | ZH161 | 828.09 ± 45.46a |  |  |  |  |
|  |  | CK | 829.97 ± 57.70a |  |  |  |  |
|  |  | KO-1 | 747.88 ± 33.28a | -80.2 | -9.7 | -82.1 | -9.9 |
|  |  | KO-2 | 771.23 ± 21.86a | -56.9 | -6.9 | -58.7 | -7.1 |

**Table S**1. Continued.

| Trial ^a^ | Trait ^b^ | Line | Mean ± SD ^c^ | ±RP ^d^ | ±RP% ^e^ | ±CK ^f^ | ±CK% ^g^ |
| --- | --- | --- | --- | --- | --- | --- | --- |
| T2 | BRR | ZH161 | 79.9 ± 0.2ab |  |  |  |  |
|  |  | CK | 80.6 ± 0.3a |  |  |  |  |
|  |  | KO-1 | 80.1 ± 0.4ab | 0.2 | 0.3 | -0.5 | -0.6 |
|  |  | KO-2 | 79.8 ± 0.5b | -0.1 | -0.1 | -0.8 | -1.0 |
|  | MRR | ZH161 | 70.6 ± 0.6a |  |  |  |  |
|  |  | CK | 70.3 ± 0.4a |  |  |  |  |
|  |  | KO-1 | 71.1 ± 0.1a | 0.5 | 0.7 | 0.8 | 1.1 |
|  |  | KO-2 | 70.4 ± 0.7a | -0.2 | -0.3 | 0.1 | 0.2 |
|  | HRR | ZH161 | 40.5 ± 5.0b |  |  |  |  |
|  |  | CK | 41.6 ± 6.0b |  |  |  |  |
|  |  | KO-1 | 54.6 ± 4.7a | 14.1 | 34.8 | 13.0 | 31.1 |
|  |  | KO-2 | 53.0 ± 3.2a | 12.5 | 30.1 | 11.4 | 27.4 |
|  | HD | ZH161 | 83.4 ± 1.7a |  |  |  |  |
|  |  | CK | 81.6 ± 0.8a |  |  |  |  |
|  |  | KO-1 | 81.6 ± 0.7a | -1.8 | -2.1 | -0.0 | -0.0 |
|  |  | KO-2 | 82.6 ± 0.7a | -0.8 | -0.9 | 1.0 | 1.2 |

^a^ The T1 trial was conducted in 2019, in which 20 plants of each line were measured. The T2 trial was conducted in 2021, using a randomized complete block design with three replications.

^b^ TGW, 1,000-grain weight (g); GL, grain length (mm); GW, grain width (mm); NP, number of panicles per plant; NGP, number of grains per panicle; GY, grain yield per plot (g); BRR, brown rice recovery (%); MRR, milled rice recovery (%); HRR, head rice recovery (%); HD, heading date (d).

^c^ Numbers with different letters are significantly different at *p* < 0.05 based on the Duncan’s multiple range test.

^d^ Increase or decrease over the recipient ZH161.

^e^ Percentage increase or decrease over ZH161.

^f^ Increase or decrease over the knock-out negative control (CK).

^g^ Percentage increase or decrease over CK (%).

**Table S2.** Haplotypes of *OsPUB3* in rice germplasms.

| Haplotype | SNP1 | SNP2 | SNP3 | SNP4 | SNP5 | SNP6 | NoV 1 ^a^ | | |  |  |  | NoV 2 ^b^ | Place of the  two parents |
| --- | --- | --- | --- | --- | --- | --- | --- | --- | --- | --- | --- | --- | --- | --- |
|  | 97 | 438 | 755 | 830 | 1546 | 1995 | Jap | Ind | Int | Aus | Total |  |  |  |
| Hap1 | T | C | C | A | T | T | 27 | 2690 | 45 | 267 | 3029 |  | 230 | MY46 |
| Hap2 | C | T | C | A | A | C | 943 | 50 | 24 | 1 | 1018 |  | 19 | ZS97 |
| Hap3 | T | C | C | A | A | C | 471 | 4 | 104 | 1 | 580 |  | 7 |  |
| Hap4 | C | C | C | G | A | C | 43 | 1 | 2 | 0 | 46 |  | 1 |  |
| Hap5 | T | C | C | G | A | C | 12 | 0 | 0 | 0 | 12 |  | 0 |  |
| Hap6 | T | C | T | A | T | T | 0 | 5 | 0 | 0 | 5 |  | 0 |  |
| Hap7 | T | C | C | A | A | T | 2 | 0 | 1 | 0 | 3 |  | 0 |  |
| Hap8 | T | C | C | A | T | C | 0 | 1 | 1 | 0 | 2 |  | 0 |  |

**^a^** Number of varieties in 4695 rice germplasms. Jap, Japonica; Ind, Indica; Int, intermediate. **^b^** Number of varieties in 257 *indica* rice germplasms.

| Table S3. Rice germplasms used in this study. | | | | |
| --- | --- | --- | --- | --- |
| Name | Origin | Type ^a^ | Haplotype of *OsPUB3* | Functional type of *GSE5* ^b^ |
| Dular | India | Ex | Hap1 | GSE5 |
| Gu 2725 | Cuba | Ex | Hap1 | GSE5 |
| HPA 74 | India | Ex | Hap1 | GSE5 |
| BG 915 | Sri Lanka | Ex | Hap1 | GSE5 |
| BR 601-3-3-2-4 | Bangladesh | Ex | Hap1 | GSE5 |
| MA HNAN THU KA | Burma | Ex | Hap1 | GSE5 |
| X72-7-1 | Burma | Ex | Hap1 | GSE5 |
| 5173 | Columbia | Ex | Hap1 | GSE5 |
| AMISTAD 82-8 | Cuba | Ex | Hap1 | GSE5 |
| C.CORTO 1 | Cuba | Ex | Hap1 | GSE5 |
| SELECCION VG-5 | Cuba | Ex | Hap1 | GSE5 |
| GIZA 181 | Egypt | Ex | Hap1 | GSE5 |
| CSR 5 | India | Ex | Hap1 | GSE5 |
| K 428-25 | India | Ex | Hap1 | GSE5 |
| NDR 89 | India | Ex | Hap1 | GSE5 |
| CIMANUK | Indonesia | Ex | Hap1 | GSE5 |
| Shuiyuan299 | North Korea | Ex | Hap1 | GSE5 |
| KHARA GANJA(ACC76363） | Pakistan | Ex | Hap1 | GSE5 |
| 75-1-120 | Philippines | Ex | Hap1 | GSE5 |
| BG 1165-1 | Sri Lanka | Ex | Hap1 | GSE5 |
| CSR 12 | India | Ex | Hap1 | GSE5 |
| CSR 13 | India | Ex | Hap1 | GSE5 |
| CSR 9 | India | Ex | Hap1 | GSE5 |
| NDR 4012 | India | Ex | Hap1 | GSE5 |
| PR32-PD47-PD4 | India | Ex | Hap1 | GSE5 |
| RP 1017-76-1-3-2 | India | Ex | Hap1 | GSE5 |
| B 6397F-MR-7-5M-1-1 | Indonesia | Ex | Hap1 | GSE5 |
| GANJAY(ACC76349) | Pakistan | Ex | Hap1 | GSE5 |
| BASMATI 685 | Pakistan | Ex | Hap1 | GSE5 |
| PANAMA 1537 | Panama | Ex | Hap1 | GSE5 |
| PR 23631-98 | Philippines | Ex | Hap1 | GSE5 |
| Pratao Precoce | Philippines | Ex | Hap1 | GSE5 |
| SINNA SIVAPPU | Sri Lanka | Ex | Hap1 | GSE5 |
| SINANDOMENG | Philippines | Ex | Hap1 | GSE5 |
| BERI | Philippines | Ex | Hap1 | GSE5 |
| RANGPUR (KANGPUR) | Bangladesh | Ex | Hap1 | GSE5 |
| NEANG CHHOUK | Cambodia | Ex | Hap1 | GSE5 |
| 368 | Pakistan | Ex | Hap1 | GSE5 |
| CHAH NONG NGHE AN | Vietnam | Ex | Hap1 | GSE5 |
| CHIEM BAC | Vietnam | Ex | Hap1 | GSE5 |
| Table S3 (continued) | | | | |
| Name | Origin | Type | Haplotype of *OsPUB3* | Functional type of *GSE5* |
| RE BAU | Vietnam | Ex | Hap1 | GSE5 |
| LAI TRANG | Vietnam | Ex | Hap1 | GSE5 |
| RE CHANH | Vietnam | Ex | Hap1 | GSE5 |
| JHALI | Nepal | Ex | Hap1 | GSE5 |
| Basmati 370 | Pakistan | Ex | Hap1 | GSE5 |
| Harwa | India | Ex | Hap1 | GSE5 |
| Kajaru | India | Ex | Hap1 | GSE5 |
| kasalath | India | Ex | Hap1 | GSE5 |
| TM 10265 | India | Ex | Hap1 | gse5 |
| CSR 11 | India | Ex | Hap1 | gse5 |
| KALING A2 | India | Ex | Hap1 | gse5 |
| OR 79-21 | India | Ex | Hap1 | gse5 |
| BAHBUTONG | Indonesia | Ex | Hap1 | gse5 |
| BATANG PANE | Indonesia | Ex | Hap1 | gse5 |
| CIPUNEGARA | Indonesia | Ex | Hap1 | gse5 |
| Sanjiang | Japan | Ex | Hap1 | gse5 |
| Milyang 70 | North Korea | Ex | Hap1 | gse5 |
| 97A-M54 | Philippines | Ex | Hap1 | gse5 |
| ITA 304 | Nigeria | Ex | Hap1 | gse5 |
| BG 170 | Sri Lanka | Ex | Hap1 | gse5 |
| habataki | Japan | Ex | Hap1 | gse5 |
| AKHNI SAIL | Bangladesh | Ex | Hap1 | gse5 |
| VL DHAN 16 | India | Ex | Hap1 | gse5 |
| BAU HUONG DOONG | Vietnam | Ex | Hap1 | gse5 |
| QUCM | Vietnam | Ex | Hap1 | gse5 |
| SINABA | Philippines | Ex | Hap1 | gse5 |
| Himdhan | India | Ex | Hap1 | gse5 |
| Koyalari | India | Ex | Hap1 | gse5 |
| SHINKWANG | Korea | Ex | Hap1 | gse5 |
| Yangdao 2 | Sri Lanka | Ex+ | Hap1 | GSE5 |
| IR36 | Philippines | Ex+ | Hap1 | GSE5 |
| IR24 | Philippines | Ex+ | Hap1 | GSE5 |
| IR64 | Philippines | Ex+ | Hap1 | GSE5 |
| Milyang 46 | North Korea | Ex+ | Hap1 | GSE5 |
| IR8 | Philippines | Ex+ | Hap1 | gse5 |
| Liantangzao 4 | Jiangxi | ImV | Hap1 | gse5 |
| Xiangwanxian 9 | Hunan | ImV | Hap1 | GSE5 |
| Jiayu 948 | Zhejiang | ImV | Hap1 | GSE5 |
| Ezao 14 | Hubei | ImV | Hap1 | GSE5 |
| Ezao 18 | Hubei | ImV | Hap1 | GSE5 |
| Table S3 (continued) | | | | |
| Name | Origin | Type | Haplotype of *OsPUB3* | Functional type of *GSE5* |
| Ganzaoxian 26 | Jiangxi | ImV | Hap1 | GSE5 |
| Ganzaoxian 37 | Jiangxi | ImV | Hap1 | GSE5 |
| Jiayu 935 | Zhejiang | ImV | Hap1 | GSE5 |
| Gengxian 89 | Guangdong | ImV | Hap1 | GSE5 |
| Texianzhan 13 | Guangdong | ImV | Hap1 | GSE5 |
| Tiandongxiang | Guangxi | ImV | Hap1 | GSE5 |
| Xiangzaoxian 13 | Hunan | ImV | Hap1 | GSE5 |
| Xiangzaoxian 14 | Hunan | ImV | Hap1 | GSE5 |
| Xiangzaoxian 31 | Hunan | ImV | Hap1 | GSE5 |
| Yuexiangzhan | Guangdong | ImV | Hap1 | GSE5 |
| Zaogui 1 | Guangxi | ImV | Hap1 | GSE5 |
| Zhong 86-44 | Zhejiang | ImV | Hap1 | GSE5 |
| Zhongyouzao 3 | Zhejiang | ImV | Hap1 | GSE5 |
| Xiangaizao 7 | Hunan | ImV | Hap1 | GSE5 |
| Guangchangai | Guangdong | ImV | Hap1 | GSE5 |
| Hong 410 | Fujian | ImV | Hap1 | GSE5 |
| Xiangzaoxian 3 | Hunan | ImV | Hap1 | GSE5 |
| Nanjing 16 | Jiangsu | ImV | Hap1 | GSE5 |
| Yangdao 6 | Jiangsu | ImV | Hap1 | GSE5 |
| Yangfuxian 5 | Jiangsu | ImV | Hap1 | GSE5 |
| Yangfuxian 6 | Jiangsu | ImV | Hap1 | GSE5 |
| Yuxian 3 | Henan | ImV | Hap1 | GSE5 |
| Ganwanxian 14 | Jiangxi | ImV | Hap1 | GSE5 |
| Ganwanxian 19 | Jiangxi | ImV | Hap1 | GSE5 |
| Ganwanxian 30 | Jiangxi | ImV | Hap1 | GSE5 |
| Qiguizao25 | Guangdong | ImV | Hap1 | GSE5 |
| Qilisimiao | Guangdong | ImV | Hap1 | GSE5 |
| Sanerai | Guangdong | ImV | Hap1 | GSE5 |
| Xianxiaozhan | Guangdong | ImV | Hap1 | GSE5 |
| Xiangwanxian 13 | Hunan | ImV | Hap1 | GSE5 |
| Xiangwanxian 17 | Hunan | ImV | Hap1 | GSE5 |
| Shuangzhuzhan | Guangdong | ImV | Hap1 | GSE5 |
| Yuchi 231-8 | Hunan | ImV | Hap1 | GSE5 |
| 5450 | Jiangxi | ImV | Hap1 | GSE5 |
| M112 | Jiangxi | ImV | Hap1 | GSE5 |
| Tuanjie 1 | Guangxi | ImV | Hap1 | GSE5 |
| Dongtingwanxian | Hunan | ImV | Hap1 | GSE5 |
| Xiangwanxian 1 | Hunan | ImV | Hap1 | GSE5 |
| Ce 64-7 | Hunan | ImV | Hap1 | GSE5 |
| Ce 64 | Hunan | ImV | Hap1 | GSE5 |
| Table S3 (continued) | | | | |
| Name | Origin | Type | Haplotype of *OsPUB3* | Functional type of *GSE5* |
| Shuhui 527 | Sichuan | ImV | Hap1 | GSE5 |
| Fuhui 838 | Sichuan | ImV | Hap1 | GSE5 |
| Minghui 86 | Fujian | ImV | Hap1 | GSE5 |
| Fengaizhan 1 | Guangdong | ImV | Hap1 | GSE5 |
| Zhong 9B | Zhejiang | ImV | Hap1 | GSE5 |
| Jin 23 B | Hunan | ImV | Hap1 | GSE5 |
| Huazhan | Zhejiang | ImV | Hap1 | GSE5 |
| Erjiufeng | Zhejiang | ImV | Hap1 | gse5 |
| Xiangzaoxian 6 | Hunan | ImV | Hap1 | gse5 |
| Zhe733 | Zhejiang | ImV | Hap1 | gse5 |
| Zhe 852 | Zhejiang | ImV | Hap1 | gse5 |
| Zhefu 802 | Zhejiang | ImV | Hap1 | gse5 |
| Ezao 6 | Hubei | ImV | Hap1 | gse5 |
| Qishanzhan | Guangdong | ImV | Hap1 | gse5 |
| Xiangzaoxian 17 | Hunan | ImV | Hap1 | gse5 |
| Xiangzaoxian 19 | Hunan | ImV | Hap1 | gse5 |
| Xiangzaoxian 24 | Hunan | ImV | Hap1 | gse5 |
| Aijiaonante | Guangdong | ImV | Hap1 | gse5 |
| Ainanzao 1 | Zhejiang | ImV | Hap1 | gse5 |
| Chaoyang 1 | Zhejiang | ImV | Hap1 | gse5 |
| Qinglian 16 | Jiangsu | ImV | Hap1 | gse5 |
| Xiujiangzao 9 | Jiangxi | ImV | Hap1 | gse5 |
| Xiangaizao 10 | Hunan | ImV | Hap1 | gse5 |
| Longge 113 | Fujian | ImV | Hap1 | gse5 |
| Huaai 15 | Hubei | ImV | Hap1 | gse5 |
| Aizizhan | Guangxi | ImV | Hap1 | gse5 |
| Chaoyangzao 18 | Guangdong | ImV | Hap1 | gse5 |
| Lunanzao 1 | Sichuan | ImV | Hap1 | gse5 |
| Zhongganzao | Zhejiang | ImV | Hap1 | gse5 |
| Erjiuqing | Zhejiang | ImV | Hap1 | gse5 |
| Xianfeng 1 | Zhejiang | ImV | Hap1 | gse5 |
| Guangnongai 1 | Guangdong | ImV | Hap1 | gse5 |
| Guangluai 4 | Guangdong | ImV | Hap1 | gse5 |
| Qingganhuang | Zhejiang | ImV | Hap1 | gse5 |
| Zhuguang 23 | Anhui | ImV | Hap1 | gse5 |
| Guichao 2 | Guangdong | ImV | Hap1 | gse5 |
| Shuanggui 1 | Guangdong | ImV | Hap1 | gse5 |
| Zhenzhuai | Guangdong | ImV | Hap1 | gse5 |
| Hongmeizao | Guangdong | ImV | Hap1 | gse5 |
| Zhaiyeqing 8 | Guangdong | ImV | Hap1 | gse5 |
| Table S3 (continued) | | | | |
| Name | Origin | Type | Haplotype of *OsPUB3* | Functional type of *GSE5* |
| Kejiexuan 17 | Guangdong | ImV | Hap1 | gse5 |
| Yuanfengzao | Zhejiang | ImV | Hap1 | gse5 |
| Simei 2 | Zhejiang | ImV | Hap1 | gse5 |
| Xiangzaoruo | Hunan | ImV | Hap1 | gse5 |
| Zhongzao 33 | Zhejiang | ImV | Hap1 | gse5 |
| Yuxian 5 | Henan | ImV | Hap1 | gse5 |
| Teqing | Guangdong | ImV | Hap1 | gse5 |
| Nanjing 11 | Jiangsu | ImV | Hap1 | gse5 |
| Aituogu 151 | Sichuan | ImV | Hap1 | gse5 |
| Lushuang 1011 | Sichuan | ImV | Hap1 | gse5 |
| Dijiaowujian | Taiwan | ImV | Hap1 | gse5 |
| Taizhongzailai 1 | Taiwan | ImV | Hap1 | gse5 |
| Bayiwan | Jiangxi | ImV | Hap1 | gse5 |
| Aijiaobaimizi | Fujian | ImV | Hap1 | gse5 |
| 754 | Jiangxi | ImV | Hap1 | gse5 |
| Nantehao | Jiangxi | ImV | Hap1 | gse5 |
| Guangchang 13 | Guangdong | ImV | Hap1 | gse5 |
| Lucaihao | Fujian | ImV | Hap1 | gse5 |
| Guangyexian | Anhui | Lr | Hap1 | GSE5 |
| Shanlanzhan | Guangdong | Lr | Hap1 | GSE5 |
| Shuangkanghong | Hunan | Lr | Hap1 | GSE5 |
| Fanganzao | Fujian | Lr | Hap1 | GSE5 |
| Yintiaozhan | Henan | Lr | Hap1 | GSE5 |
| Huangbansuo | Yunnan | Lr | Hap1 | GSE5 |
| Dishuigu | Guizhou | Lr | Hap1 | gse5 |
| Wubaili | Hubei | Lr | Hap1 | gse5 |
| Shuibawang | Hubei | Lr | Hap1 | gse5 |
| Yiluxiang | Anhui | Lr | Hap1 | gse5 |
| Maotudimeng | Anhui | Lr | Hap1 | gse5 |
| Puningmalongya | Guangdong | Lr | Hap1 | gse5 |
| Lengshuizhan | Guizhou | Lr | Hap1 | gse5 |
| Gaoganhe | Hubei | Lr | Hap1 | gse5 |
| Machengjiangxizao | Hubei | Lr | Hap1 | gse5 |
| Yapozhan | Hunan | Lr | Hap1 | gse5 |
| Daozhouzao | Hunan | Lr | Hap1 | gse5 |
| Changuzao | Hunan | Lr | Hap1 | gse5 |
| Qishizao | Sichuan | Lr | Hap1 | gse5 |
| Taizhonghongxuguzi | Taiwan | Lr | Hap1 | gse5 |
| Maoxiangzhan | Chongqing | Lr | Hap1 | gse5 |
| Wujiezaodao | Anhui | Lr | Hap1 | gse5 |
| Table S3 (continued) | | | | |
| Name | Origin | Type | Haplotype of *OsPUB3* | Functional type of *GSE5* |
| Jiugongji | Jiangxi | Lr | Hap1 | gse5 |
| Sanlicun | Anhui | Lr | Hap1 | gse5 |
| Baijiaoe | Anhui | Lr | Hap1 | gse5 |
| Muqiuchui | Anhui | Lr | Hap1 | gse5 |
| Nibuzhan | Anhui | Lr | Hap1 | gse5 |
| Zhuchaxian | Anhui | Lr | Hap1 | gse5 |
| Wanminuo | Guizhou | Lr | Hap1 | gse5 |
| Ziguilengshuigu | Hubei | Lr | Hap1 | gse5 |
| Tiejiaozhan | Hunan | Lr | Hap1 | gse5 |
| Langanzhan | Hunan | Lr | Hap1 | gse5 |
| Dayexiaoxianggu | Shanxi | Lr | Hap1 | gse5 |
| Zaomiaosi | Shanxi | Lr | Hap1 | gse5 |
| Dalangan | Sichuan | Lr | Hap1 | gse5 |
| Lengshuizao | Yunnan | Lr | Hap1 | gse5 |
| Xiaomaweizhan | Chongqing | Lr | Hap1 | gse5 |
| Qinggangu | Guizhou | Lr | Hap1 | gse5 |
| Hongmidongzhan | Hubei | Lr | Hap1 | gse5 |
| Chihe | Hunan | Lr | Hap1 | gse5 |
| Youzhan | Hunan | Lr | Hap1 | gse5 |
| Yinghongke | Jiangsu | Lr | Hap1 | gse5 |
| Bayuebai | Jiangxi | Lr | Hap1 | gse5 |
| Yingguzhan | Jiangxi | Lr | Hap1 | gse5 |
| Niantianshi | Zhejiang | Lr | Hap1 | gse5 |
| Zigangu | Yunnan | Lr | Hap1 | gse5 |
| Xigu | Yunnan | Lr | Hap1 | gse5 |
| Babaomi | Yunnan | Lr | Hap1 | gse5 |
| Baiganlufeng | Yunnan | Lr | Hap1 | gse5 |
| Bairizao | Yunnan | Lr | Hap1 | gse5 |
| Niumaoxiangzao | Yunnan | Lr | Hap1 | gse5 |
| Lengshuibaigu | Yunnan | Lr | Hap1 | gse5 |
| JW 60 | India | Ex | Hap2 | GSE5 |
| Nova 66 CI9481 | USA | Ex | Hap2 | gse5 |
| Zhongyouzao 81 | Zhejiang | ImV | Hap2 | GSE5 |
| Jiahezaozhan | Fujian | ImV | Hap2 | GSE5 |
| Zhou 903 | Zhejiang | ImV | Hap2 | GSE5 |
| Bo B | Guangxi | ImV | Hap2 | GSE5 |
| Xieqingzao | Anhui | ImV | Hap2 | GSE5 |
| II-32B | Hunan | ImV | Hap2 | GSE5 |
| Jiayu 293 | Zhejiang | ImV | Hap2 | gse5 |
| Wenxuanqing | Zhejiang | ImV | Hap2 | gse5 |
| Table S3 (continued) | | | | |
| Name | Origin | Type | Haplotype of *OsPUB3* | Functional type of *GSE5* |
| Xiangzaoxian 1 | Hunan | ImV | Hap2 | gse5 |
| Guangjie 9 | Guangdong | ImV | Hap2 | gse5 |
| Zhe 76-1 | Zhejiang | ImV | Hap2 | gse5 |
| Zhenshan 97 | Zhejiang | ImV | Hap2 | gse5 |
| Zhongzao 39 | Zhejiang | ImV | Hap2 | gse5 |
| Zhongjiazao 17 | Zhejiang | ImV | Hap2 | gse5 |
| Mancang 515 | Fujian | ImV | Hap2 | gse5 |
| V20B | Hunan | ImV | Hap2 | gse5 |
| Zhaodaowen | Sichuan | Lr | Hap2 | gse5 |
| IB 28 | Burundi | Ex | Hap3 | GSE5 |
| IB 42 | Burundi | Ex | Hap3 | GSE5 |
| RT 1031-69 | Congo | Ex | Hap3 | GSE5 |
| T 23 | India | Ex | Hap3 | GSE5 |
| PADI SEGUTUK | Indonesia | Ex | Hap3 | GSE5 |
| RATRIA(ACC28500) | Pakistan | Ex | Hap3 | GSE5 |
| BINICOL | Philippines | Ex | Hap3 | GSE5 |
| PIRURUTONG | Philippines | Ex | Hap4 | gse5 |

**^a^** ImV, Improved variety in China; Lr, Landrace in China; Ex, Exotic germplasm; Ex+, Exotic germplasm widely used in China. ^b^ *GSE5*: functional; *gse5*: non-functional.

**Table S4.** Primers used in this study.

| Name | Sequence (5'-3') | Purpose |
| --- | --- | --- |
| Cri-PUB3 | F: TGTGTGCGTCCGACGGGGAGCTGCTC | Knock-out vector construction |
|  | R: AAACGAGCAGCTCCCCGTCGGACGCA |  |
| CP-PUB3 | F: ATCCTCTAGAGTCGAACTTCCTTCATTGCCAGGG | Complementary vector construction |
|  | R: CCAAGCTTGCATGCCCTAGTCCACTCTAACCGTCACC |  |
| Hyg | F: GTTTATCGGCACTTTGCATCG | Transgene detection |
|  | R: GGAGCATATACGCCCGGAGT |  |
| Seq-PUB3 | F: GCCTCGAGATGGACGCGAAC | Sequencing and genotyping |
|  | R: TCTGGCTCTTCGTCGGT |  |
| S3900 | F: CCAATCGTCGCAATCACTCC | Sequencing for *OsPUB3* |
|  | R: CCCTTTCCAGCCAATGCATC |  |
| GSE5-Del | F: CTTTCAACTCCGATTGGCAT | Genotyping for *GSE5* |
|  | R: GTAGGGAAGGAGCTGCATGA |  |
